# Supplementary material for: Whole genome deconvolution unveils Alzheimer’s resilient epigenetic signature
Source: Nat Commun. 2023 Aug 16;14:4947. doi: 10.1038/s41467-023-40611-4 (PMC10432546; doi:10.1038/s41467-023-40611-4)
Supplement: Supplementary file 1 — Supplementary Information [file 41467_2023_40611_MOESM1_ESM.pdf]

Supplementary Information: Whole genome deconvolution unveils Alzheimer's resilient epigenetic signature.

Eloise Berson[1,2,3+], Anjali Sreenivas[1,2], Thanaphong Phongpreecha[1,2,3], Amalia Perna[1], Fiorella C. Grandi[4,5,6], Lei Xue[2,3,7], Neal G. Ravindra[1,2,3], Neelufar Payrovnaziri[2,3,7], Samson Mataraso[2,3,7], Yeasul Kim[2,3,7], Camilo Espinosa[2,3,7], Alan L. Chang[2,3,7], Martin Becker[2,3,7], Kathleen S. Montine[1], Edward J. Fox[1], Howard Y. Chang[8,9], M. Ryan Corces[4,5,6], Nima Aghaeepour[2,3,7*] & Thomas J. Montine[1*]

Affiliations:

[1]Department of Pathology, Stanford University; Stanford, CA, United States

[2]Department of Anesthesiology, Perioperative, and Pain Medicine, Stanford University; Stanford, CA, United States

[3]Department of Biomedical Data Science, Stanford University; Stanford, CA, United States

[4]Gladstone Institute of Neurological Disease, San Francisco, CA, United States

[5]Gladstone Institute of Data Science and Biotechnology, San Francisco, CA, United States.

[6]Department of Neurology, University of California San Francisco, San Francisco, CA, United States.

[7]Department of Pediatrics, Stanford University; Stanford, CA, United States

[8]Center for Personal Dynamic Regulomes, Stanford University School of Medicine, Stanford, CA, USA

[9]Howard Hughes Medical Institute, Stanford University School of Medicine, Stanford, CA, USA

*: Co-senior authors

+: Corresponding author eloiseb@stanford.edu

Supplementary Figures

	Female	Male	Age (+/- sd)	White	Black/African American	Asian
NC	2	2	80.33 +/- 12.96	3	1	0
RAD	6	6	82.25 +/- 7.63	10	1	1
ADD	8	11	89.95 +/- 6.08	17	0	2

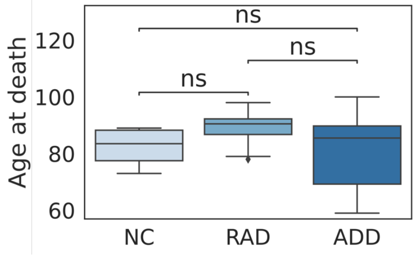

Supplementary Figure 1: Dataset overview: sex, age, and ethnicity across groups. ADD = Alzheimer's disease dementia, RAD = resilient to Alzheimer's disease, NC = normal control, ns = not significant. The box plot shows the median (middle line), interquartile range (bottom and upper edges), and the minimum and maximum values of the distribution (whiskers). P-values were derived using the adjusted two-sided Wilcoxon's test.

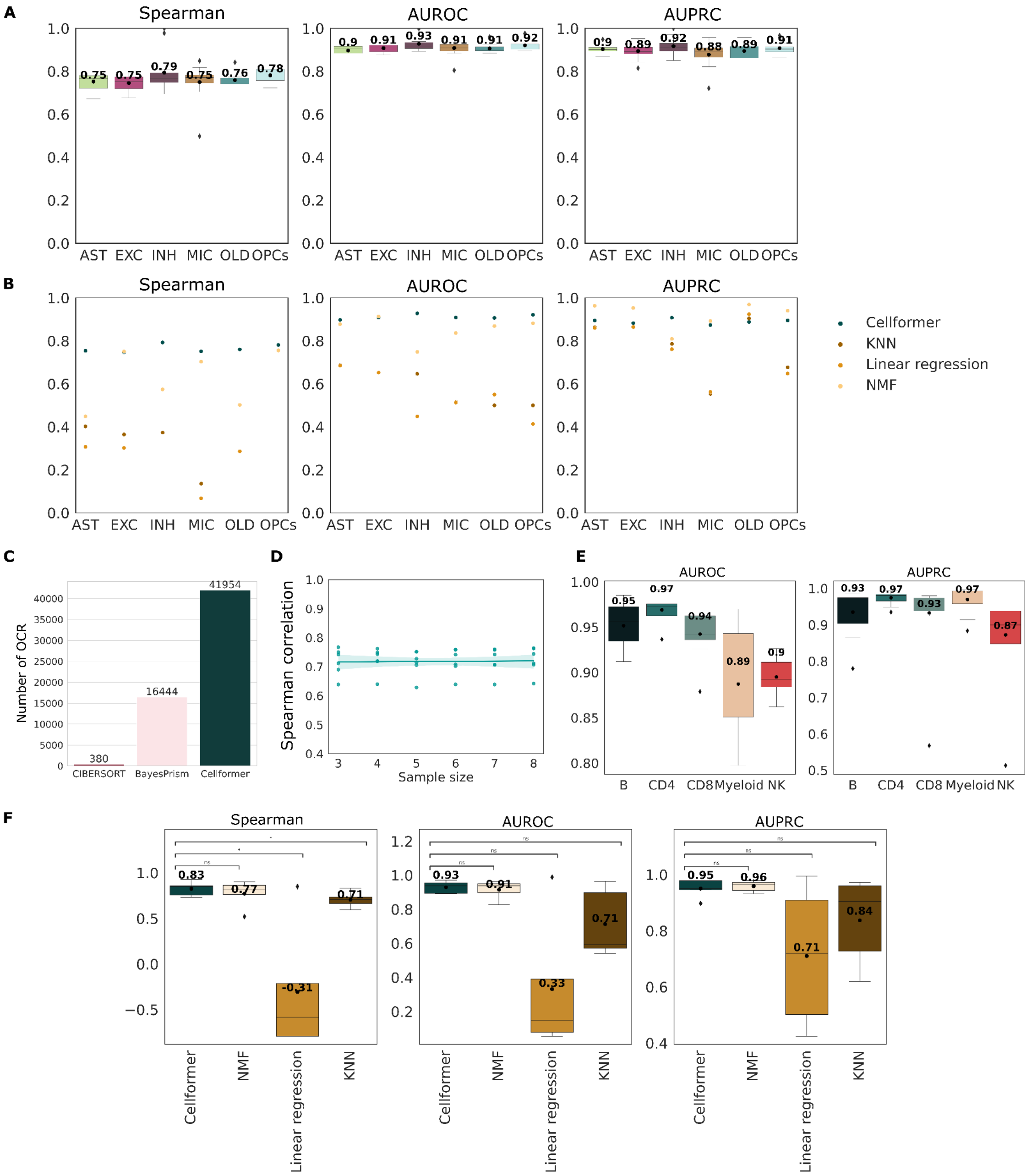

Supplementary Figure 2: Cellformer applied to PBMC ATAC-seq data from different cohorts. A. Leave-one-out cross-validated Cellformer performance stratified by cell type (n=13 samples). Cellformer output preserves cell type signature across 6 cell types: astrocytes (AST), microglia (MIC), oligodendrocytes (OLD) and oligodendrocyte progenitor cells (OPCs) and 2 major classes of neurons, excitatory (EXC) and inhibitory (INH). **B.** Cellformer outperforms the baseline models across cell types. **C.** Barplot comparing the number of generated features among CIBERSORT[1], BayesPrism[2] and Cellformer. **D.** Cellformer performance when trained using varying numbers of snATAC-seq samples. To limit confounders, we restricted our analysis to samples from the same brain region, SMTG. Cellformer is weakly impacted by sample size (Two-sided Krustal-Wallis P-value 0.98). The error band represents the 95% confidence interval. **E.** Cellformer successfully deconvoluted PBMC in-silico bulk ATAC-seq data (n=18 samples), predicting cell type-specific expression of 5 main cell types (B cell, T-cell-CD4+ (CD4), T cell-CD8+ (CD8), Myeloid and NK cells), removing cell types present in less than 1% per samples[3]. **F.** Cellformer outperforms the baseline models with a minimal cross-sample variation, with a significantly higher Spearman correlation than Linear regression and KNN across the different cell types (n=6). All box plots show the median (middle line), interquartile range (bottom and upper edges), and the minimum and maximum values of the distribution (whiskers). (P-values derived using two-sided Wilcoxon's tests after multi-testing correction)

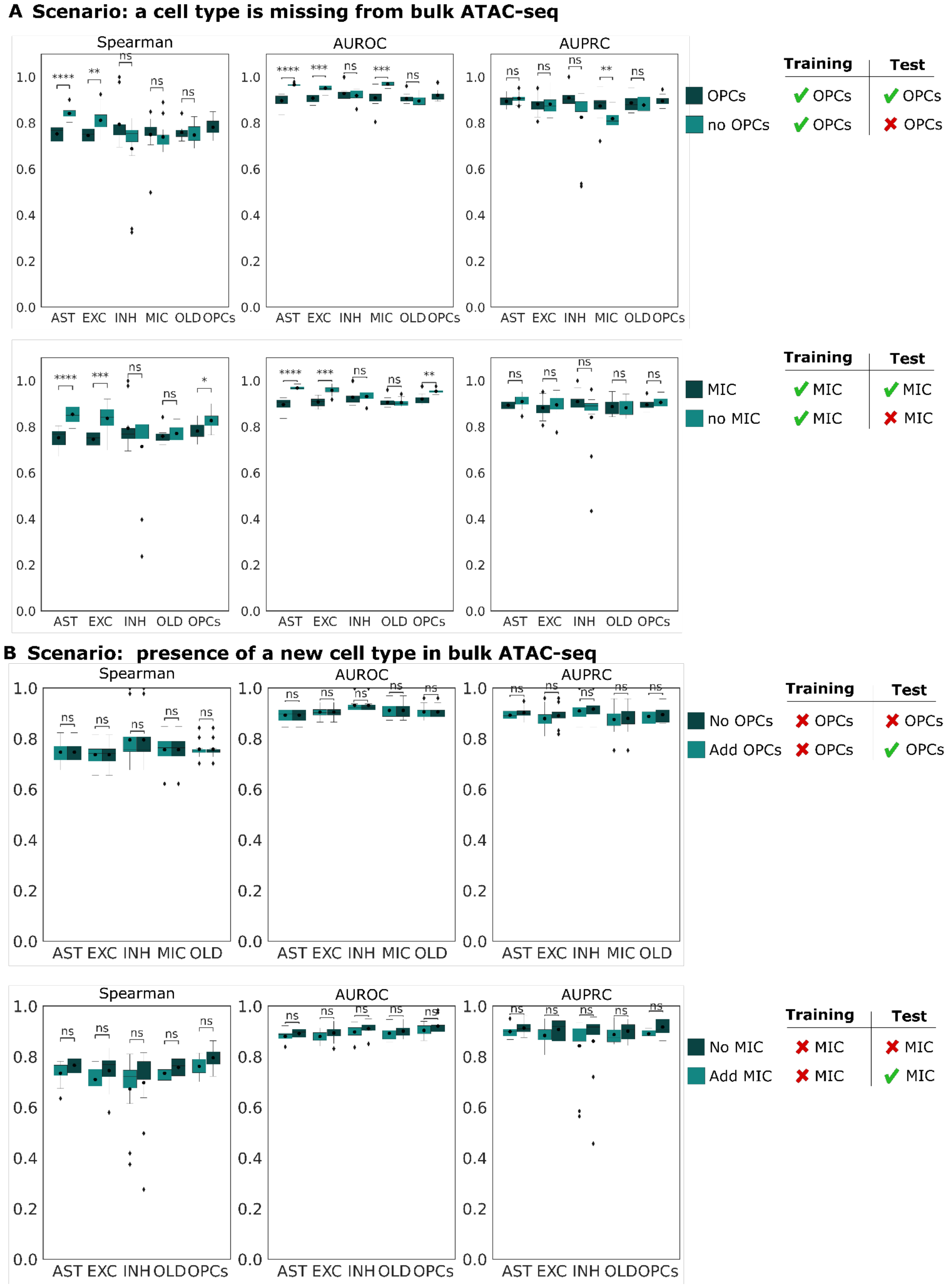

Supplementary Figure 3: Cellformer evaluation in real-life scenarios. A. Simulation of a real-life scenario where a cell type is absent in bulk tissue. Boxplots illustrate Cellformer performance when two rare cell types, OPCs and MIC, are missing from the pseudo bulk samples used to test the model. Top panel represents the absence of OPCs, bottom panel represents the absence of MIC. P-values were derived using two-sided Wilcoxon's tests. **B.** Simulation of a real-life scenario where a new cell type, previously unseen by the model, emerges in bulk tissue. Boxplots illustrate Cellformer performance when two rare cell types, OPCs and MIC, are intentionally removed from the synthetic pseudo bulk samples during training and added at testing. Top panel represents the absence of OPCs; bottom panel represents the absence of MIC. (n=13 samples). P-values were derived using two-sided Wilcoxon's tests. All box plots show the median (middle line), interquartile range (bottom and upper edges), and the minimum and maximum values of the distribution (whiskers). "*": P-value<0.05, "**":P-value<0.01, "***": P-value<0.001, "****": P-value<0.0001.

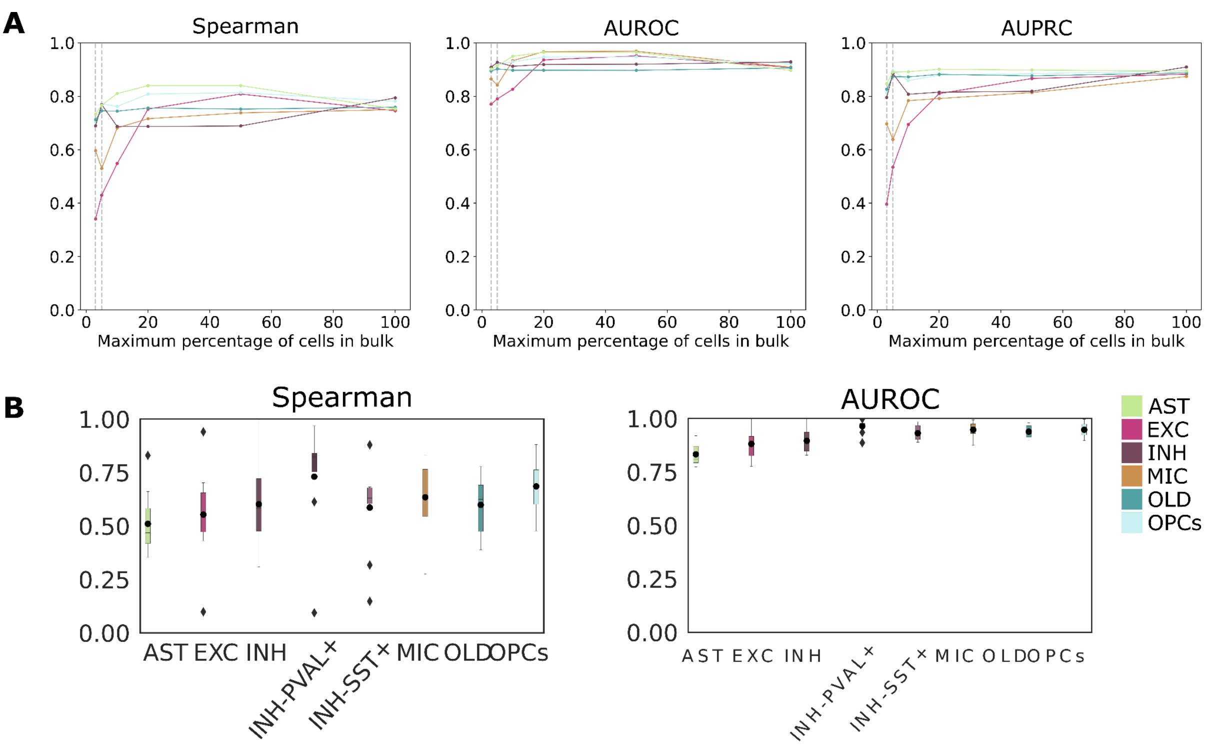

Supplementary Figure 4: Cellformer evaluation on synthetic datasets. A. Cellformer performance evaluated using synthetic pseudo bulk data, with varying percentages of cells per cell type. **B.** Cellformer performance when trained to deconvolute bulk ATAC-seq data at a lower resolution (n=13). All box plots show the median (middle line), interquartile range (bottom and upper edges), and the minimum and maximum values of the distribution (whiskers).

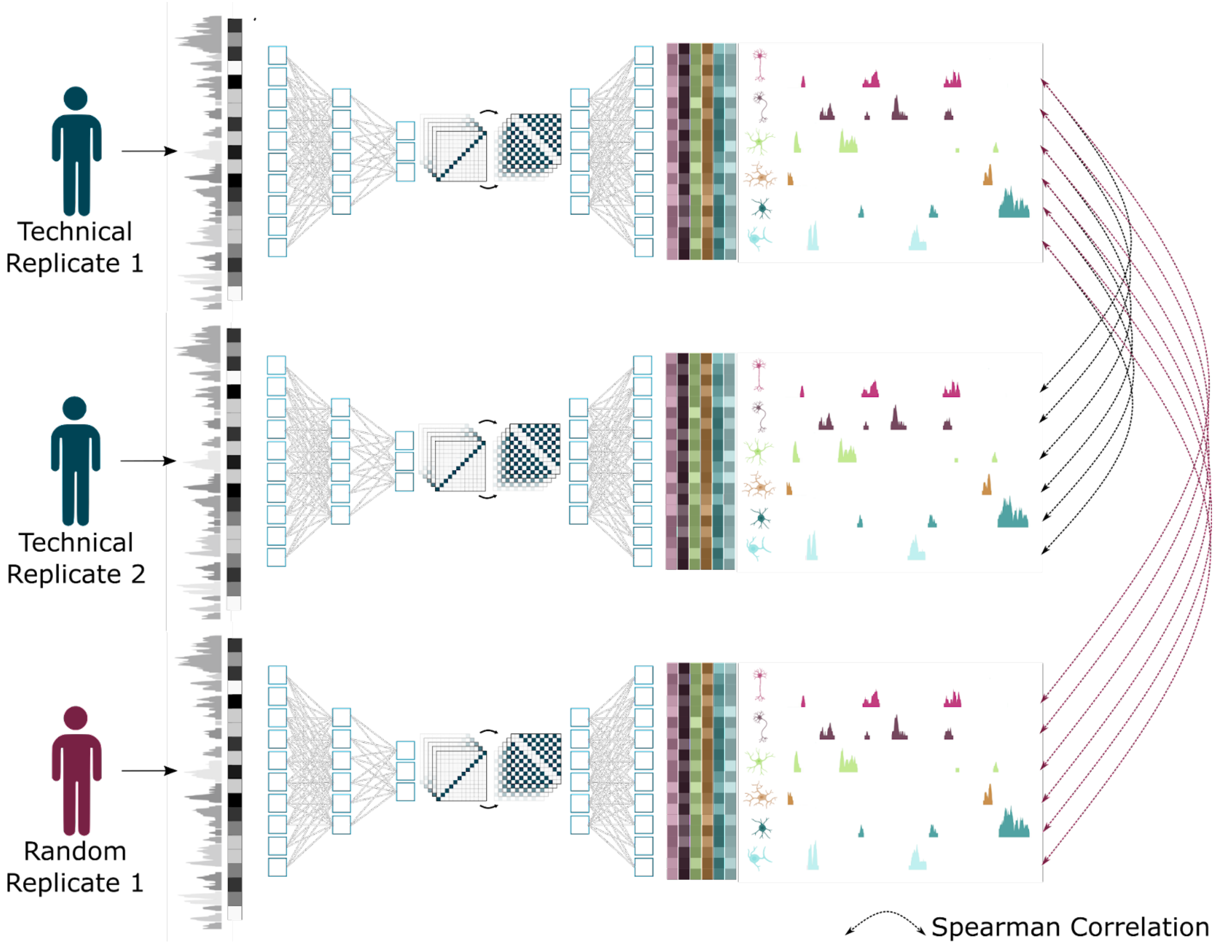

Supplementary Figure 5: Model consistency evaluation. Quality of model predictions was assessed by comparing technical replicate cell type-specific expression using Spearman correlation between cell type expression (created with Biorender).

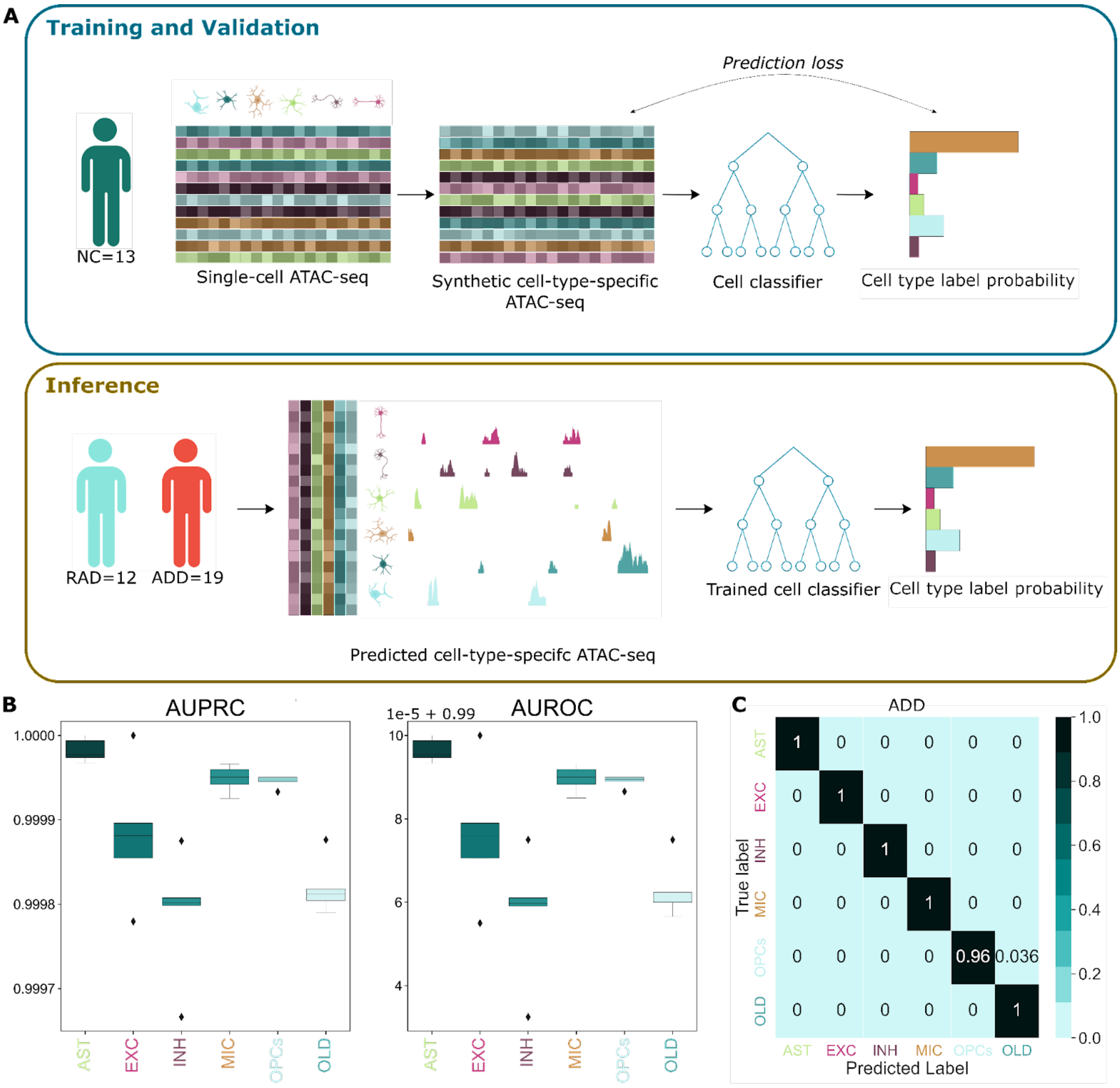

Supplementary Figure 6: Cell classifier measured cell type signature preservation of Cellformer **A.** XGBoost model was trained to predict the cell type from synthetic cell type-specific ATAC-seq data created from real single-cells. Once trained and validated, the cell classifier was applied to Cellformer predictions from RAD and ADD (created with Biorender.com). **B**. Performance of the cell classifier across cross-validation iterations stratified by cell type (n=36 samples). **C**. Performance of the trained classifier applied to ADD deconvoluted cell type-specific expression. All box plots show the median (middle line), interquartile range (bottom and upper edges), and the minimum and maximum values of the distribution (whiskers).

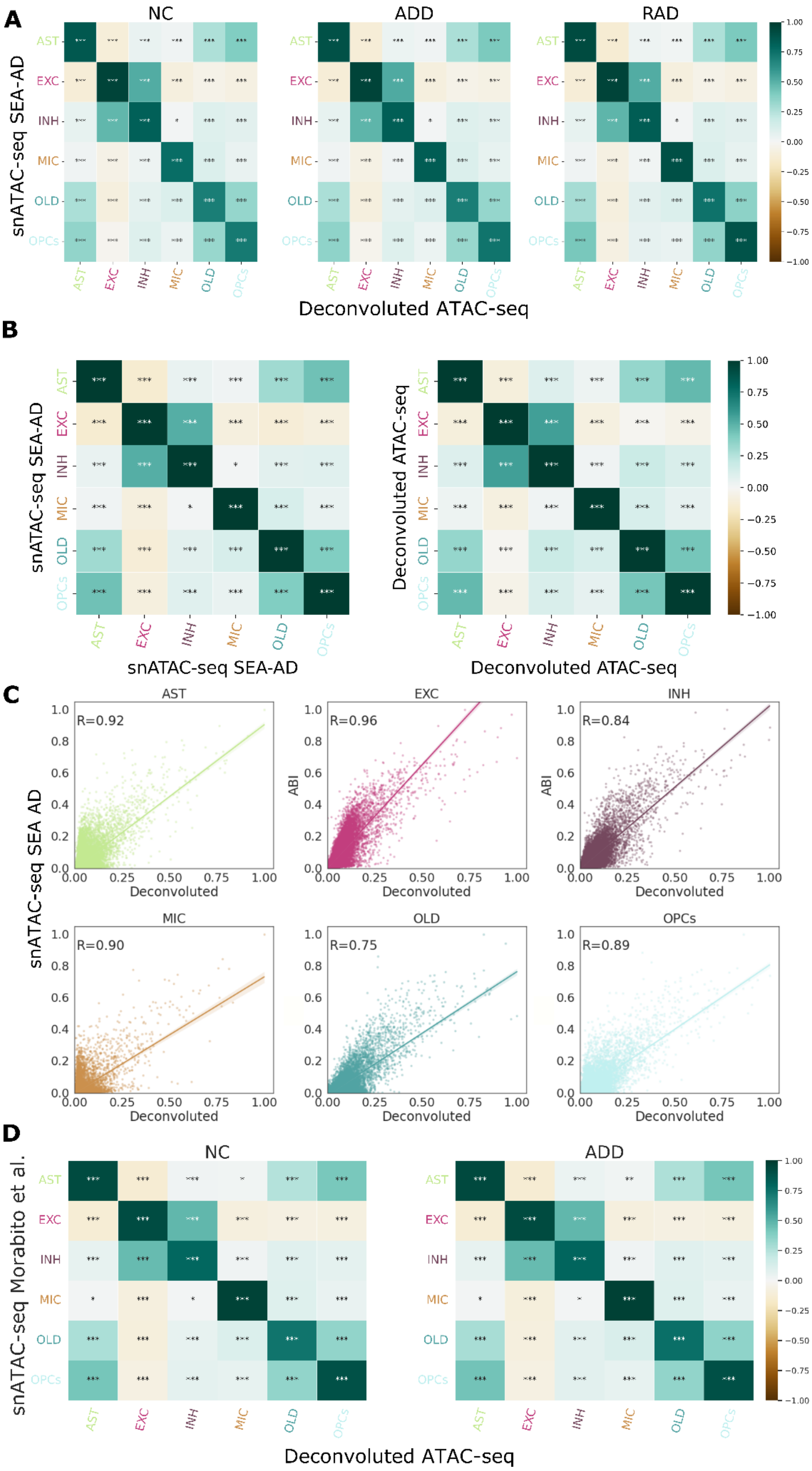

Supplementary Figure 7: Validation of the model output using an external single nucleus ATAC-seq dataset from SMTG. A. Correlation matrices between Cellformer outputs and single nucleus ATAC-seq from the SEA-AD database, stratified by phenotype. P-values were derived using two-sided spearman correlations. **B.** snATAC-seq and deconvoluted ATAC-seq mean profile autocorrelation matrices. P-values were derived using two-sided spearman correlations. **C.** Regression plot of cell type-specific OCR between deconvoluted and an external dataset in RAD, stratified per cell type. **D.** Correlation matrices between Cellformer outputs and single nucleus ATAC-seq from[4]. P-values were derived using two-sided spearman correlations. "*": P-value<0.05, "**":P-value<0.01, "***": P-value<0.001, "****": P-value<0.0001.

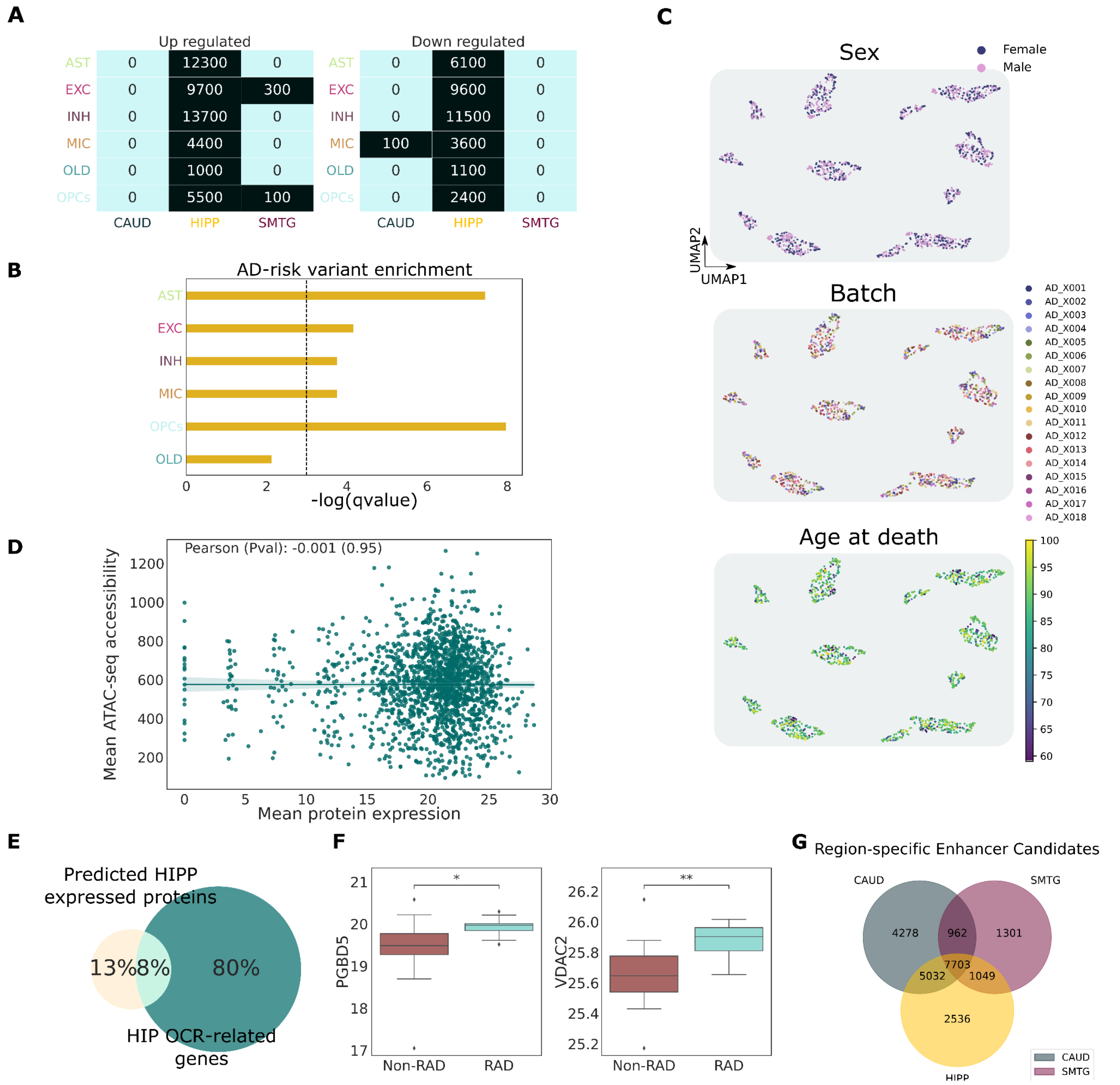

Supplementary Figure 8: Cellformer output acts as deconfounded in-silico single nucleus. A. Cellformer generated cell type-specific expression restoring known biological signature of ADD**. B.** ADD differentially regulated OCR enrichment in ADD GWAS. Our approach enabled the retrieval of already-known signals. Enrichment of GWAS genetic candidates in ADD-specific OCR was assessed using the Fisher test. **C.** Minimal impact of biological and technical confounders on cell type-specific expression. **D.** Correlation between the mean protein expression and ATAC-seq accessibility from NC in HIPP. We used promoters associated with protein-coding genes. The error band represents the 95% confidence interval. P-value was derived using a two-sided spearman correlation test. **E.** The overlap between OCR-related genes and expressed proteins in HIPP. **F.** Proteomic expression levels of PGBD5 and VDAC2 which are also upregulated in RAD at the epigenetic level (n=36 samples). The box plot shows the median (middle line), interquartile range (bottom and upper edges), and the minimum and maximum values of the distribution (whiskers). P-values were derived using two-sided Wilcoxon's tests after multi-testing correction**. G.** Number of predicted enhancers per brain region using ABC model[5].

Supplementary Tables

	Normal Control	RAD	ADD
B Score	< 3	> 2	> 2
C score	0	> 1	> 1
Cognitive Diagnosis	No dementia within 2 year of death	No dementia within two year of death	Dementia
Vascular brain injury	None	None	None
Lewy Body	None	None	None
LATE-NC	< 1	< 1	< 1

Supplementary Table 1: Cases selection criteria for bulk ATAC-seq[6] and single-cell ATAC-seq from the Seattle Alzheimer's Disease Brain Cell Atlas (SEA-AD) Cohort[7].

Supplementary References

1. Newman, A. M. *et al.* Robust enumeration of cell subsets from tissue expression profiles. *Nat. Methods* **12**, 453–457 (2015).
2. Chu, T., Wang, Z., Pe'er, D. & Danko, C. G. Cell type and gene expression deconvolution with BayesPrism enables Bayesian integrative analysis across bulk and single-cell RNA sequencing in oncology. *Nat Cancer* **3**, 505–517 (2022).
3. Hartmann, F. J. *et al.* Comprehensive Immune Monitoring of Clinical Trials to Advance Human Immunotherapy. *Cell Rep* **28**, 819-831.e4 (2019).
4. Morabito, S. *et al.* Single-nucleus chromatin accessibility and transcriptomic characterization of Alzheimer's disease. *Nat Genet* **53**, 1143–1155 (2021).
5. Fulco, C. P. *et al.* Activity-by-contact model of enhancer–promoter regulation from thousands of CRISPR perturbations. *Nat. Genet.* **51**, 1664–1669 (2019).
6. Corces, M. R. *et al.* Single-cell epigenomic analyses implicate candidate causal variants at inherited risk loci for Alzheimer's and Parkinson's diseases. *Nat. Genet.* **52**, 1158–1168 (2020).
7. Travaglini, K. J. *et al.* A multimodal atlas of the molecular and cellular changes to cortex driven by Alzheimer's disease. *Alzheimers Dement* **18**, (2022).
