# Supplementary material for: Whole genome deconvolution unveils Alzheimer’s resilient epigenetic signature
Source: Nat Commun. 2023 Aug 16;14:4947. doi: 10.1038/s41467-023-40611-4 (PMC10432546; doi:10.1038/s41467-023-40611-4)
Supplement: Supplementary file 3 — Reporting Summary [file 41467_2023_40611_MOESM3_ESM.pdf]

nature portfolio

Corresponding author(s): Dr. Eloise Berson

Last updated by author(s): 2023/07/21

Reporting Summary

Nature Portfolio wishes to improve the reproducibility of the work that we publish. This form provides structure for consistency and transparency in reporting. For further information on Nature Portfolio policies, see our Editorial Policies and the Editorial Policy Checklist.

Statistics

For all statistical analyses, confirm that the following items are present in the figure legend, table legend, main text, or Methods section.

n/a	Confirmed	
☐	☒	The exact sample size (*n*) for each experimental group/condition, given as a discrete number and unit of measurement
☐	☒	A statement on whether measurements were taken from distinct samples or whether the same sample was measured repeatedly
☐	☒	The statistical test(s) used AND whether they are one- or two-sided<br>*Only common tests should be described solely by name; describe more complex techniques in the Methods section.*
☐	☒	A description of all covariates tested
☐	☒	A description of any assumptions or corrections, such as tests of normality and adjustment for multiple comparisons
☐	☒	A full description of the statistical parameters including central tendency (e.g. means) or other basic estimates (e.g. regression coefficient) AND variation (e.g. standard deviation) or associated estimates of uncertainty (e.g. confidence intervals)
☐	☒	For null hypothesis testing, the test statistic (e.g. *F*, *t*, *r*) with confidence intervals, effect sizes, degrees of freedom and *P* value noted<br>*Give P values as exact values whenever suitable.*
☐	☒	For Bayesian analysis, information on the choice of priors and Markov chain Monte Carlo settings
☐	☒	For hierarchical and complex designs, identification of the appropriate level for tests and full reporting of outcomes
☐	☒	Estimates of effect sizes (e.g. Cohen's *d*, Pearson's *r*), indicating how they were calculated

Our web collection on statistics for biologists contains articles on many of the points above.

Software and code

Policy information about availability of computer code

Data collection	No software was used for data collection
Data analysis	For future research, all custom code used in this work code, processed data and additional metadata have been made publicly available at (https://github.com/elo-nsrb/Cellformer) and https://doi.org/10.5281/zenodo.8175353. The following packages were used: Python 3 (version 3.9.7) with PyTorch (version 1.10.0); Scikit-learn (version 1.0.1), asteroid (0.5.2), and GSEAPY (version 1.0.3); R (version 4.2.2) with ArchR (R version 4.2.2), Chipseeker (version 1.36.0); FeatureCount (version 2.0.3).

For manuscripts utilizing custom algorithms or software that are central to the research but not yet described in published literature, software must be made available to editors and reviewers. We strongly encourage code deposition in a community repository (e.g. GitHub). See the Nature Portfolio guidelines for submitting code & software for further information.

Data

Policy information about availability of data

All manuscripts must include a data availability statement. This statement should provide the following information, where applicable:

- Accession codes, unique identifiers, or web links for publicly available datasets
- A description of any restrictions on data availability
- For clinical datasets or third party data, please ensure that the statement adheres to our policy

All data supporting the findings described in this manuscript are publicly available. Bulk ATAC-seq and single-cell ATAC-seq from control individuals were previously collected and annotated 26 accessible through GEO accession (GSE147672) https://www.ncbi.nlm.nih.gov/geo/query/acc.cgi?acc=GSE147672. Additional single-nucleus ATAC-seq data and raw and processed bulk ATAC-seq from ADD and RAD are available through GEO accession (GSE226529) (https://www.ncbi.nlm.nih.gov/geo/query/acc.cgi?acc=GSE226529) and Dryad (DOI: 10.5061/dryad.2fqz612t0). Validation of the model was performed using processed snATAC-seq from 40,41 available at (http://portal.brain-map.org/explore/seattle-alzheimers-disease and https://www.synapse.org/#!Synapse:syn22079621/wiki/603535. Processed PBMC ATAC-seq data are accessible at https://github.com/GreenleafLab/ArchR_2020).

Human research participants

Policy information about studies involving human research participants and Sex and Gender in Research.

Reporting on sex and gender	Donors were 46% female and 54% male. Gender information was not reported.
Population characteristics	Post-mortem human brain samples were used in this work from Alzheimer disease dementia (n=19), resilient(n=12) and control (n=4). These samples were taken from individuals ranging from 59 to 100 years old (mean of 83.9) from primarily Caucasian ancestry. These individuals were cognitively assessed.
Recruitment	Participants were research volunteers in the Stanford, Arizona, or University of Washington Alzheimer's Disease Research Center, who consented to donate their brains for research following each institutions IRB-approved protocol. Bias may exist due to regional or socioeconomic factors that constrain the patient populations at these facilities which could bias towards an over-representation of caucasian individuals. No other self-selection biases are expected. Individuals' brain samples from both ATAC-seq datasets were carefully filtered according to clinical diagnosis of cognitive status proximate to death and assessment of Alzheimer's disease neuropathologic change and other neuropathologic comorbidities. Resilient cases were defined as individuals without dementia at their most recent clinical research evaluation within 2 years of death, and neuropathologic findings of B score >2 and C score > 1 but without vascular brain injury or Lewy body disease, and LATE neuropathologic change stage of 0 or 1.
Ethics oversight	Post-mortem brain samples were collected with approved consent and overseen by the relevant institutional review boards of Stanford University, the University of Washington, and Banner Health

Note that full information on the approval of the study protocol must also be provided in the manuscript.

Field-specific reporting

Please select the one below that is the best fit for your research. If you are not sure, read the appropriate sections before making your selection.

☒ Life sciences ☐ Behavioural & social sciences ☐ Ecological, evolutionary & environmental sciences

For a reference copy of the document with all sections, see nature.com/documents/nr-reporting-summary-flat.pdf

Life sciences study design

All studies must disclose on these points even when the disclosure is negative.

Sample size	Sample size was based on the available biological material. However, sufficient samples were used to allow statistical measures of reproducibility across multiple biological donors in all cases.
Data exclusions	Sequencing data that did not pass pre-established quality control filters was excluded from analysis.
Replication	Replication across independant biological samples was the primary metric for reproducibility of sequencing data. results from all sequencing runs were included in our analysis. For machine learning work, leave one out cross validation was used. See (Corces et al. 2020) for further information about data collection.
Randomization	During nuclei isolation tissue samples were randomized into batches to avoid batch effects from nuclei isolation. During bulk ATAC-seq, and scATAC-seq library construction, randomized batches were also used. For machine learning work, leave one out cross validation was used.
Blinding	All brain tissue nuclei isolation as well as scATAC-seq and bulk ATAC-seq data generation were carried out in a blinded manner. using coded samples so investigators were blinded to group allocation during data collection. In data analysis, blinding was not performed after the

collection of the ATAC-seq data, as cohort and label of each sample have been defined.

Reporting for specific materials, systems and methods

We require information from authors about some types of materials, experimental systems and methods used in many studies. Here, indicate whether each material, system or method listed is relevant to your study. If you are not sure if a list item applies to your research, read the appropriate section before selecting a response.

Materials & experimental systems

n/a	Involved in the study
☒	☐ Antibodies
☒	☐ Eukaryotic cell lines
☒	☐ Palaeontology and archaeology
☒	☐ Animals and other organisms
☒	☐ Clinical data
☒	☐ Dual use research of concern

Methods

n/a	Involved in the study
☒	☐ ChIP-seq
☒	☐ Flow cytometry
☒	☐ MRI-based neuroimaging
